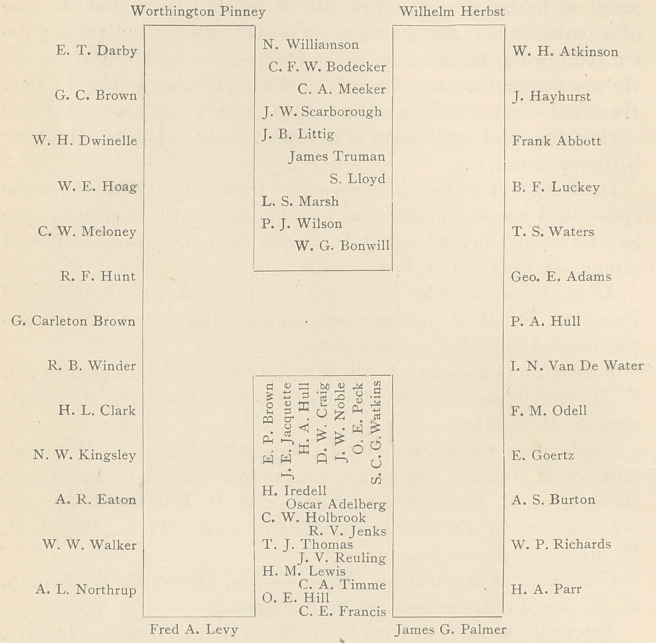# The Herbst Clinics in America

**Published:** 1886-11

**Authors:** 


### THE HERBST CLINICS IN AMERICA.

REPORTED EXPRESSLY FOR THE INDEPENDENT PRACTITIONER.

(Continued from page 582.)

On the evening of Tuesday, July 22d, certain members of the New Jersey State Dental Society gave a dinner in honor of Dr. Wilhelm Herbst and other distinguished visitors. Dr. Fred A. Levy occupied the chair. The dinner, which was given at the Coleman House, Asbury Park, N. J., was a brilliant success in every respect.

Upon the back of the elaborate *menu* appeared a large letter H, an exact copy of the arrangement of the table, with the names printed in the positions occupied by everyone present, as follows:

Dr. Fred A. Levy asked all the gentlemen to rise and drink to the health of Dr. Wm. Herbst.

Dr. Herbst answered in German, which was translated by Dr. C. F. W. Bödecker. He thanked the society for the unexpected acknowledgment, but more particularly for the great interest manifested at the clinic, which, as he remarked, gave him more pleasure than anything else.

Dr. Levy then proposed a toast to the dental profession at large, and called upon Dr. Wm. H. Dwinelle to respond.

Dr. Dwinelle spoke at some length, highly complimenting Dr. Herbst, and concluded his remarks by speaking of the dignity of the profession. The chairman then proposed a toast to the dentists of New York, and called upon Dr. N. W. Kingsley.

Dr. Kingsley responded, and in concluding his remarks called attention to the fact that that day, the 22d of July, was the sixtieth birthday of Dr. Wm. H. Dwinelle, the announcement being received with great applause.

The chairman then proposed the health of the dentists of Pennsylvania, and called upon Dr. E. T. Darby.

Dr. Darby spoke in a very complimentary way of Dr. Herbst, and hoped that when he returned he would feel that he had the good wishes of every American dentist, as he had been treated here like a professional brother. Dr. Darby then expressed the hope that the dentists of Germany would recognize the fact that the dental profession of this country has recognized in Dr. Herbst a gentleman of modesty, and a genius. (Cheers.) Dr. Darby then spoke of the dentists of Pennsylvania, and especially of Philadelphia, in connection with dental education.

Dr. Levy then proposed the health of the dentists of Maryland, and called upon Dr. R. B. Winder.

Dr. Winder, in his remarks, endorsed all that had been said by Dr. Darby in regard to Dr. Herbst, and then spoke of Baltimore as the cradle of dental education, mentioning at some length the difficulties which Dr. Chapin A. Harris had to encounter.

The chairman then proposed a toast to the dentists of Philadelphia, and called upon Dr. James Truman.

Dr. Truman congratulated the society upon the great success of the evening, and remarked that all he could say to Dr. Herbst he had expressed a few evenings since, at a dinner given him by the dentists of Philadelphia.

The chairman then called upon Dr. O. E. Hill to speak for the dentists of Brooklyn.

Dr. Hill, after responding to the toast, said he was glad that there was such a country as Germany, to produce such a genius as she has sent to us, and he therefore proposed a toast to the dentists of Germany.

Dr. Herbst again thanked the society for the honors shown him, but remarked that he would regard them as compliments paid to professional friends in his own country. He then remarked that formerly dental education in Germany had been very much neglected. A dental student was required to study two years in a college where everything was taught, *except dentistry.* He remarked that he had graduated under the same curriculum, and when he passed his examination, graduated with honor, but he had, up to that time, not known how a gold filling was inserted in the cavity of a tooth. After that he settled in Bremen, and bought all the latest American instruments, but was unable to make any use of them. When he saw the beautiful work which from time to time came to him from this country, he was stimulated to produce like results in any way he could. He knew the American proverb, "Help yourself," and out of his struggles the rotary method came into existence. He freely declared that if he had known how to insert a filling by the American system, he would never have thought to experiment in this direction, and he is of the opinion that his method in the hands of American dentists would soon be brought to great perfection.

The chairman then proposed a toast to the rising generation of dentists, and called upon Dr. W. W. Walker.

Dr. Walker was pleased to pay homage to Dr. Herbst and his baby (the rotation method), and very glad that he had been elected an honorary member of the New York State Dental Society.

The chairman then proposed a toast to the honorary members of the society, and called upon Dr. Bonwill.

Dr. W. G. A. Bonwill, after saying a few words in response to the toast, remarked that he was glad to see that the Americans honored a foreigner in this way. He regarded Dr. Herbst as a mechanical genius, and he had met only very few of them in the dental profession. In speaking of his method, Dr. Bonwill could not say whether the invention was valuable or not, until he had tried it. From what he had seen he was very favorably impressed, although the method was in direct opposition to his system of operating, but whether the new method would be universally adopted or not it was impossible to say. He then presented Dr. Herbst with one of his dental and surgical engines and mechanical mallet, under the condition that Dr. Herbst will give it as faithful a trial as he himself would the new method.

The chairman then proposed a toast to the New Jersey State Dental Society, and called upon Dr. Hayhurst.

Dr. Hayhurst at some length described the good work which, during the sixteen years of its existence, had been done by the society, and was glad that it had welcomed Dr. Herbst in so cordial a manner.

The chairman then proposed a toast to the other distinguished guests, and called upon Dr. Atkinson.

Dr. Wm. H. Atkinson had heard of Dr. Herbst first through Dr. Bödecker, whose judgment he highly respected, but he had withheld his judgment until he had an operation performed in his own mouth, and by which the truth was revealed to him that the Herbst method stands *par excellence* as the exponent of the law of the adaptation of the gold to the walls of a cavity.

The chairman then expressed the hope that the society might see all the faces again next year, to which Dr. Wm. H. Dwinelle responded, principally confining his remarks to honorable dental practitioners who had passed away, but whose names will live forever. In this connection he mentioned Chapin A. Harris, Elisha Townsend, E. B. Gardette, Eleazer Parmly, Solyman Brown, Jehiel Parmly, and others, after which the meeting adjourned.

CLINIC GIVEN JULY 23D, FOR THE SAME SOCIETY.

Dr. Herbst lined some cavities with gold for filling with amalgam, in the following manner: A large cylinder of Wolrab's gold was compressed between the fingers and dipped into a very thin solution of gum-copal in sulphuric ether, which is used for the purpose of preventing the mercury of the amalgam from uniting with the gold.

When this compressed cylinder has been moistened with the liquid, the surplus is pressed out with the fingers, the ether allowed to evaporate, and then by means of a piece of cotton, the gold is pressed against the labial wall of the cavity in the tooth to be filled with amalgam. The gold in this manner can be thoroughly condensed by means of a rotating burnisher in the engine, and will not alter the shape of the cavity. When ordinary round undercuts have been made, the amalgam will be held in position without any trouble. Dr. Herbst exhibited many teeth which had been filled in this way, several months ago, and showed no signs of discoloration, but presented the most natural appearance, although their labial walls were very thin, so much so that the gold was visible through the enamel. He then explained the making of instruments, matrices and other appliances, such as were exhibited in the trays of his cabinet.

The president, Dr. W. Pinney, then called the meeting to order, and after some routine business had been transacted, the privilege of the floor was given to Dr. Herbst, who was interpreted by Dr. Bödecker. He said:——

Mr. President and Gentlemen, I am very glad to have this opportunity to say a few words which I wanted to express last evening.

Gentlemen, Dr. Bonwill very kindly made me a present of his dental engine and mallet on condition that I shall practice with it and see what can be done, and this I promise to do. But, gentlemen, I say here openly that if I had had the skill and the training that Dr. Atkinson, Dr. Bonwill, Dr. Brown, Dr. Webb, Dr. Varney, Dr. Bödecker, and others, I should never have attempted to invent my rotation method.

I know that by means of the electrical mallet and the Bonwill mechanical mallet you can achieve very great results. I have seen a patient of Dr. Bödecker who had several large fillings that were made with the Bonwill mechanical mallet, which were very beautiful. I do not know whether Dr. Bonwill or any other gentleman here has had the two methods used in his mouth, but I am sure that any one who has will have noticed a very marked difference in the sensation produced; and I think we should consider the comfort of our patients, as well as our own. Even if an operation could be performed with the mechanical mallet, or with the electrical mallet, in about the same time and with as little labor as it can be done by the rotation method, still the patients will be much better satisfied if they are subjected to less pain and annoyance; and you know the mallet always is a more or less disagreeable instrument to experience in the introduction of gold.

I accept the present of Dr. Bonwill with gratitude, and I shall esteem it very highly, especially as the gentleman from whom it comes is known in Germany and Europe as one of the most ingenious and skillful operators in his profession. Therefore, I again thank him for the present he has kindly made me. And again my very best thanks for what this society has done for me, and the reception you have given me. I shall never forget the few days I have spent with you.

*Dr. S. C. G. Watkins*—Mr. President, Dr. Herbst has said that he would like to hear from some one who has experienced in his mouth both the Bonwill mallet and the Herbst rotary method. The first operation with a stone burnisher that was ever done in

America, I believe, was done in my mouth, by Dr. Bödecker. It was the filling of a cavity in a wisdom tooth, which was very sensitive. The doctor used different mallets, the Bonwill among others, and their effect was very unpleasant; but the rotary motion of this stone burnisher I never felt at all. There was no pain whatever from its use, but the effect of the mallets was terrible. It was a great relief to substitute the rotating blood-stone which Dr. Wheeler, of Albany, had made.

Since Dr. Herbst has been in this country, he has, it seems, absorbed the attention and thoughts of dentists through this section entirely. They have forgotten everyone else in honoring Dr. Herbst. But there is at least one other member of our profession who deserves a great deal of credit for bringing this method to our attention, for bringing Dr. Herbst to this country, and exciting an interest that will doubtless lead to further experiments and improvements. I refer to Dr. Bödecker. I think this society ought to pass a vote of thanks to Dr. Bödecker for the great service he has rendered, and the trouble and expense he has had in bringing this matter to the attention of the profession. Therefore, Mr. President, I move you that this society tender to Dr. Bödecker a vote of thanks, for the very great interest he has taken in bringing this matter before the profession.

The motion was carried unanimously.

Dr. Bödecker, in responding, said: Gentlemen, I am obliged for the sentiment you have expressed, but I believe it is the duty of every dental practitioner carefully to examine, and, if found worthy, to adopt anything that may be of value to his brethren, wherever he finds it, and to preserve and present it to his professional brothers.

I saw this rotation method about two years ago, when it was very nearly on the eve of death. At that time I believed it to be a valuable invention, and that a great deal of good would come from it. So I tried my best to save it to the world. Whatever I have done has been, not for myself or any single individual, but with a view to benefit the dental profession at large.

In response to the remarks of Dr. Herbst, Dr. Bonwill said: Mr. President and Gentlemen, I wish to say that, in return for the gift which I have presented to Dr. Herbst, I have to thank him for a set of his instruments which he has kindly tendered to me, and I

promise him that I will, so far as I know how, use those instruments and test them thoroughly in introducing gold in the manner that he has done in our presence.

I have never doubted that gold could be impacted in this way. Any mechanic who ever saw a piece of tin or sheet iron spun up with a simple burnisher would know at once that the metal assumes a new character, and is capable of welding in that way.

While this method of introducing gold may possibly be much more pleasant to the patient, that is not the only point to be considered; diminishing the disagreeable sensation of the instrument is not the only way to save the patient and to eliminate human suffering. The saving of time also tends to that end, and is of great importance to the operator. While we consider the welfare of our patients, we must not forget ourselves. In reducing the time required for these operations three-fourths, I made a great advance in the way of saving time for the patient as well as the operator.

This rotation method is the outgrowth of a keen, mechanical mind, environed and stimulated as his has been.

I thank Dr. Herbst again for the set of instruments which he has presented to me, and which I will give a fair trial.

Dr. G. Carleton Brown then proposed Dr. William Herbst, of Bremen, Germany, for honorary membership in the society.

On motion, the Secretary was authorized to cast the ballot in favor of Dr. William Herbst, who was declared duly elected an honorary member.

### CLINIC GIVEN JULY 26TH, AT THE OFFICE OF DR. BÖDECKER.

Mrs. H—— was in the chair, for whom Dr. Herbst contoured the right lower canine, the cavity involving one-third of the mesial, one-third of the distal and a portion of the cutting edge of the tooth. A shellac matrix, with three pieces of steel spring, was employed during the introduction of the gold. The matrix was made as follows: A piece of shellac, the size of a pigeon's egg, was softened and pressed behind the lingual surface, and a little over the cutting edges of the six front teeth. It was then removed, cooled in water, and two pieces of steel watch spring were inserted by heating them over an alcohol flame, and then putting them between the approximate surfaces of the eye teeth, lateral, and the first bicuspid, and into the shellac in such a manner that the anterior edge of the spring did not quite come up to the labial surface of the teeth. After these two pieces were inserted in the shellac, the whole of the matrix was replaced and pressed firmly against the tooth, while the steel spring pieces were bent into the desired position. When cold, the matrix was removed, and a third piece of steel spring was adjusted to correspond to the cutting edge of the eye tooth. The matrix was then readjusted and, when cold, taken away again, when all the surplus shellac adhering to the steel spring, as well as that which had been pressed into the cavity of the tooth, was carefully removed by means of cold excavators. Dr. Herbst again called attention to the fact that, whenever shellac matrices with steel springs are used the steel should be perfectly cleaned from surplus shellac, and the first layer of gold ought always to cover the entire surface in such a manner that the rotating instrument will not be able to touch any of it, and thus incorporate it into the first layer of gold.

The cavity, which was very large, required one hour and fifteen minutes' time for the introduction of the gold. The operation was witnessed by Drs. Ben. Lord, G. A. Mills, E. Goertz, C. H. Degenhard, and C. F. W. Bödecker. Dr. Herbst said that this was the first time he had made such a large contour operation in the mouth of a patient with gold, and he was very glad that the operation was satisfactory to himself as well as to everybody present. The gold first used in the operation was Wolrab's gold cylinders, No. 0, followed by annealed strips of No. 30 rolled gold, which adapted itself very beautifully, giving nice edges and an extremely hard and solid surface.

CLINIC GIVEN JULY 27TH, AT THE OFFICE OF DR. C. F. W. BÖDECKER.

Miss —— was in the chair, for whom Dr. Herbst filled the left upper first molar, the cavity involving the distal, the mesial and the largest portion of the grinding and lingual surface of the tooth. The operation occupied about one hour. Dr. Bödecker, at the same time, filled a left lower second molar, for a private patient, the cavity involving the mesial and grinding surfaces, using the German silver matrix and the Herbst method of filling, The operation required about forty-five minutes for the introduction of the gold. The gentlemen present at this clinic were Dr. Tennison of New York and Dr. G. H. Westlake of Virden, Ill.

(TO BE CONTINUED.)

## Figures and Tables

**Figure f1:**